# Newly diagnosed T-large granular lymphocyte Leukemia presenting with severe neutropenia: a case report

**DOI:** 10.1093/omcr/omaf170

**Published:** 2025-09-28

**Authors:** Soukaina El Asmar, Najat Lasri, Fatimazahra Lahlimi, Hicham Yahyaoui, Illias Tazi

**Affiliations:** Hematology & Bone Marrow Transplantation Department, University Hospital Mohammed VI, Faculty of Medicine and Pharmacy, Cadi Ayyad University, Avenue Ibn Sina, District Al Massira, Marrakesh 40000, Marrakesh-Safi Region, Morocco; Hematology & Bone Marrow Transplantation Department, University Hospital Mohammed VI, Faculty of Medicine and Pharmacy, Cadi Ayyad University, Avenue Ibn Sina, District Al Massira, Marrakesh 40000, Marrakesh-Safi Region, Morocco; Hematology & Bone Marrow Transplantation Department, University Hospital Mohammed VI, Faculty of Medicine and Pharmacy, Cadi Ayyad University, Avenue Ibn Sina, District Al Massira, Marrakesh 40000, Marrakesh-Safi Region, Morocco; Biological Hematology Department, Avicenne Military Hospital, Cadi Ayyad University, Marrakesh, Morocco; Hematology & Bone Marrow Transplantation Department, University Hospital Mohammed VI, Faculty of Medicine and Pharmacy, Cadi Ayyad University, Avenue Ibn Sina, District Al Massira, Marrakesh 40000, Marrakesh-Safi Region, Morocco

**Keywords:** T-cell large granular lymphocyte leukemia, neutropenia, immunosuppressive therapy, flow cytometry, cyclophosphamide

## Abstract

T-large granular lymphocyte leukemia (T-LGLL) is an uncommon chronic lymphoproliferative syndrome marked by clonal proliferation of cytotoxic (CD8+) T cells. It is usually characterized by cytopenia, particularly neutropenia, and may be associated with autoimmune disease. We report a case of a 56-year-old Moroccan female patient presenting with a 2-month history of dry eye syndrome, asthenia, and severe neutropenia. Initial investigations revealed lymphocytosis, and the peripheral blood smear revealed approximately 13% of large granular lymphocytes. Flow cytometry confirmed T-LGLL with a CD3+, CD8+, CD57+ phenotype. First-line treatment with low-dose methotrexate yielded no improvement after six months. The patient was then successfully treated with oral cyclophosphamide, with normalization of neutrophil and hemoglobin levels, and resolution of sicca symptoms. This case highlights the importance of early diagnosis and tailored immunosuppressive therapy in managing T-LGLL, particularly in patients with severe neutropenia.

## Introduction

Large granular lymphocyte leukemia (LGLL) is an uncommon lymphoproliferative disorder, accounting for approximately 2% to 5% of chronic lymphoproliferative diseases in Western populations and up to 5–6% in Eastern populations [1]. LGLL is classified into two main subtypes: T-cell large granular lymphocyte leukemia (T-LGLL), which constitutes about 85% of cases, and chronic natural killer (NK)-cell lymphocytosis, which comprises around 10% [2]. T-LGLL arises from the clonal proliferation of mature cytotoxic T cells, predominantly CD8+ cells, that exhibit an activated phenotype and cytotoxic granules. The disease is characterized by bicytopenias, particularly neutropenia and anemia, often in association with lymphocytosis. Thrombocytopenia is less common. Clinical manifestations range from asymptomatic laboratory abnormalities to severe infections and autoimmune phenomena.

A striking feature of T-LGLL is its frequent association with autoimmune disorders, especially rheumatoid arthritis. Other autoimmune conditions, such as Sjögren’s syndrome, systemic lupus erythematosus, and autoimmune thyroiditis, may also occur [3]. Although the precise mechanisms driving the disease remain under investigation, recent advances have uncovered persistent survival signaling and STAT3/STAT5 mutations in a subset of patients, implicating these pathways in disease pathogenesis [4].

Due to its indolent course, many patients with T-LGLL do not require immediate treatment. However, therapy is indicated in symptomatic individuals, particularly those with severe neutropenia, anemia requiring transfusions, or associated autoimmune disease requiring immunosuppression. Standard treatment typically involves low-dose methotrexate, cyclophosphamide, or cyclosporin A, with targeted therapies such as Alemtuzumab reserved for refractory cases [5].

We report a case of T-LGLL in a 56-year-old Moroccan woman presenting with isolated profound neutropenia and sicca symptoms, diagnosed via peripheral blood smear and immunophenotyping. This case highlights the diagnostic and therapeutic challenges in resource-limited settings and underscores the value of a structured, multidisciplinary approach to rare hematologic diseases.

## Case report

A 56-year-old woman presented to our hematology clinic with a 3-month history of progressive fatigue and sicca symptoms (dry eyes and mouth). Her medical history included well-controlled type 2 diabetes mellitus and hypertension, managed with oral medications. She had no history of recurrent infections, weight loss, or night sweats, and there was no personal or family history of hematologic malignancy or autoimmune disease.

On physical examination, the patient was afebrile and hemodynamically stable. There was no palpable lymphadenopathy, splenomegaly, or hepatomegaly. Her mucous membranes were dry, but no oral ulcers or joint abnormalities were noted.

**Figure 1:**
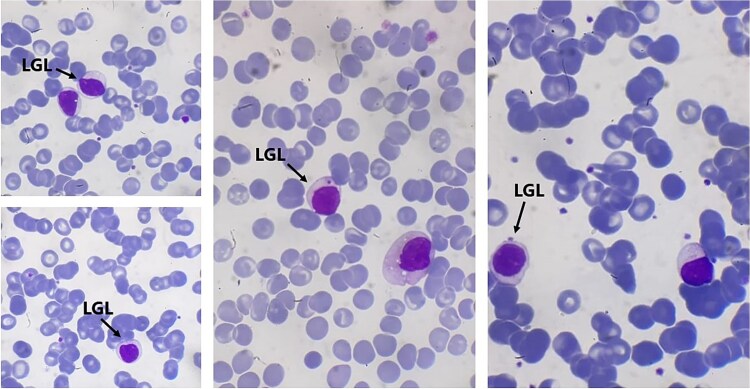
Patient’s blood smear showing large monomorphic granular lymphocytes with numerous rolled red blood cells (A, B, C and D).

Laboratory tests showed isolated lymphocytosis (6311 cells/mm^3^; reference range: 1500–4000) and bicytopenia, with severe neutropenia (137 cells/mm^3^) and mild normocytic anemia (hemoglobin 10.6 g/dL). Platelet count was within normal limits. Peripheral blood smear revealed a predominance of T-LGLL, accounting for 70% of lymphocytes. These cells were characterized by abundant pale cytoplasm with azurophilic granules and mature nuclear chromatin (Fig. 1). Red blood cells showed rouleaux formation.

Further laboratory test included vitamin B12 and folate levels, which were normal, ruling out nutritional causes of cytopenia. A comprehensive autoimmune panel, including ANA, anti-dsDNA, anti-SSA, and anti-SSB, was negative. Direct antiglobulin (Coombs) test was also negative.

Bone marrow aspiration was attempted but was technically unsuccessful. However, the core biopsy demonstrated normocellular marrow without evidence of malignancy or dysplasia. Flow cytometry performed on peripheral blood identified a clonal T-cell population expressing CD3^+^, CD4^−^, CD8^+^, CD5^+^, CD7^−^, CD57^+^, and TCR α/β^+^, consistent with a cytotoxic T-cell phenotype. This population accounted for 44% of total lymphocytes. T-cell receptor (TCR) gene rearrangement studies by PCR were requested to confirm clonality, but the test was unavailable due to technical and financial constraints.

Despite the absence of molecular confirmation, the diagnosis of T-LGLL was considered established based on the patient’s clinical presentation (neutropenia, sicca symptoms), characteristic peripheral blood smear morphology, and immunophenotypic findings on flow cytometry. This diagnostic approach is accepted in real-world clinical practice, particularly in low-resource environments.

**Figure 2:**
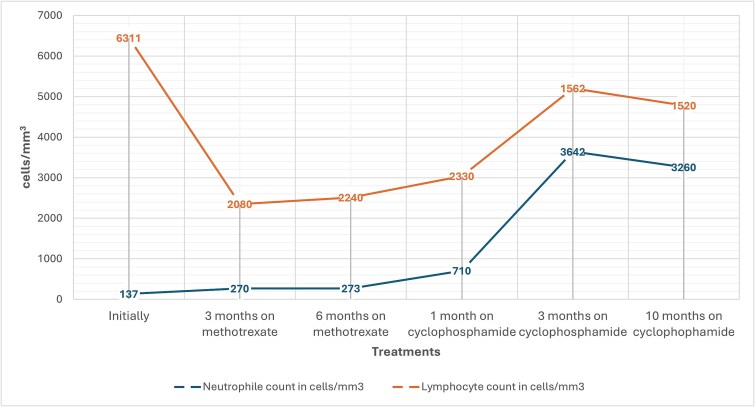
Patient’s neutrophil and lymphocyte counts on treatment.

Treatment was initiated with low-dose methotrexate (10 mg/m^2^ weekly) combined with folic acid supplementation. After 6 months, the patient showed no improvement in either clinical symptoms or hematological parameters. Consequently, therapy was switched to oral cyclophosphamide (100 mg daily). Over the next 12 months, the patient experienced resolution of fatigue and dry eyes, and her neutrophil and hemoglobin levels gradually normalized (Fig. 2). No infections or adverse drug effects were noted. At last follow-up, 10 months after completing cyclophosphamide therapy, the patient remained in hematological and clinical remission.

## Discussion

First described in 1985, LGLL is now recognized as a distinct lymphoproliferative disorder originating from mature T or NK cells [6]. The 2022 WHO classification of hematolymphoid tumors includes T-LGLL as a subtype of mature T-cell neoplasms [7]. Pathogenesis involves chronic antigenic stimulation, persistent survival signaling, and dysregulation of apoptosis. STAT3 and STAT5B mutations, present in 30–40% of patients, contribute to the pathobiology by activating downstream cytokine pathways, including IL-6, IL-15, and IFN-γ [4].

T-LGLL typically affects older adults, with a median age at diagnosis of 66 years [1]. Our patient was younger than the typical age range, reflecting the variability in presentation. While many cases are asymptomatic, symptomatic patients often present with neutropenia-related infections, anemia-related fatigue, or autoimmune symptoms. In our patient, severe neutropenia was the initial and only hematological abnormality, accompanied by sicca symptoms in the absence of autoimmune markers—an unusual constellation.

Diagnosis requires a combination of clinical, morphological, immunophenotypic, and molecular findings. On peripheral smear, LGLs appear as large lymphocytes with characteristic azurophilic granules. Flow cytometry is essential for identifying a clonal T-cell population. Our patient had the typical immunophenotype of CD3^+^, CD8^+^, CD57^+^, TCR α/β^+^ cells. Although PCR-based TCR gene rearrangement studies are valuable for confirming clonality, the diagnosis may be made without them in appropriate clinical settings, especially when other evidence supports the diagnosis [8, 9].

Treatment is generally reserved for symptomatic patients. The main indications include symptomatic cytopenias (particularly neutropenia < 500 cells/mm^3^), transfusion dependence, or autoimmune complications [3]. Immunosuppressive agents such as methotrexate, cyclophosphamide, or cyclosporin A are commonly used. Methotrexate remains the first-line therapy due to its safety profile, but response rates are modest and delayed. Cyclophosphamide has demonstrated greater efficacy in some studies, especially for cytopenias, though it carries a risk of long-term toxicity, including cytopenia and secondary malignancies [10].

Targeted therapies such as Alemtuzumab, a monoclonal antibody against CD52, have shown promise in refractory disease with an overall response rate of 60%, but are limited by cost, accessibility, and immunosuppression-associated infections [5]. JAK inhibitors like tofacitinib are under investigation but remain experimental in most settings.

Our patient failed to respond to methotrexate but achieved durable remission with cyclophosphamide, highlighting the need for individualized therapy. In resource-constrained environments, the choice of treatment must balance efficacy, safety, availability, and cost.

## Conclusion

This case underscores the diagnostic and therapeutic challenges posed by T-LGLL, especially in settings where molecular diagnostics and targeted therapies are unavailable. In patients with unexplained neutropenia, particularly when persistent and profound, T-LGLL should be considered even in the absence of autoimmune markers. A comprehensive diagnostic approach incorporating cytology, flow cytometry, and, when available, molecular testing is critical for accurate diagnosis. Immunosuppressive therapy remains the cornerstone of management, and cyclophosphamide may be a valuable second-line option when methotrexate fails. Multicenter collaboration and investment in diagnostic infrastructure are essential for improving outcomes in rare hematologic malignancies in low-resource regions.

## Consent

In accordance with Moroccan law no. 28–13, written informed consent was obtained from the patient for the publication of this report, in line with the journal’s patient consent policy.

## Guarantor

Dr Soukaina El Asmar, Hematology & Bone Marrow Transplantation Department, University Hospital Mohammed VI, Faculty of Medicine and Pharmacy, Cadi Ayyad University, Marrakesh, Morocco. Mail: s.elasmar.res@uca.ac.ma.

## References

[1] Loughran TP Jr . Clonal diseases of large granular lymphocytes. Blood. 1993;82:1–14. 10.1182/blood.V82.1.1.bloodjournal8211 8324214

[2] Rose MG, Berliner N. T-cell large granular lymphocyte leukemia and related disorders. Oncologist. 2004;9:247–58. 10.1634/theoncologist.9-3-247 15169980

[3] Moignet A, Lamy T. Latest advances in the diagnosis and treatment of large granular lymphocytic leukemia. Am Soc Clin Oncol Educ Book 2018;38:616–25. 10.1200/EDBK_200689 30231346

[4] Patnaik MM, Vallapureddy R, Lasho TL. et al. A comparison of clinical and molecular characteristics of patients with systemic mastocytosis with chronic myelomonocytic leukemia to CMML alone. Leukemia 2018;32:1850–6. 10.1038/s41375-018-0121-1 29712989

[5] Lamy T, Loughran TP. How I treat LGL leukemia. Blood. 2011;117:2764–74. 10.1182/blood-2010-07-296962 21190991 PMC3062292

[6] Loughran TP Jr, Kadin ME, Starkebaum G. et al. Leukemia of large granular lymphocytes: association with clonal chromosomal abnormalities and autoimmune neutropenia, thrombocytopenia, and hemolytic anemia. Ann Intern Med 1985;102:169–75. 10.7326/0003-4819-102-2-169 3966754

[7] Alaggio R, Amador C, Anagnostopoulos I. et al. The 5th edition of the World Health Organization classification of Haematolymphoid tumours: lymphoid neoplasms. Leukemia 2020;36:1720–48. 10.1038/s41375-022-01620-2 PMC9214472 35732829

[8] Lamy T, Moignet A, Loughran TP. Jr LGL leukemia: from pathogenesis to treatment. Blood. 2017;129:1082–94. 10.1182/blood-2016-08-692590 28115367

[9] Cheon H, Dziewulska KH, Moosic KB. et al. Advances in the diagnosis and treatment of large granular lymphocytic leukemia. Curr Hematol Malig Rep 2020;15:103–12. 10.1007/s11899-020-00565-6 32062772 PMC7234906

[10] Chin-Yee B, Suthakaran A, Hedley BD. et al. T-cell clonality testing for the diagnosis of T-cell large granular lymphocytic leukemia: are we identifying pathology or incidental clones? Int J Lab Hematol 2022;44:1115–20. 10.1111/ijlh.13949 36380468

